# Methodological Quality of User-Centered Usability Evaluation of Ambient Assisted Living Solutions: A Systematic Literature Review

**DOI:** 10.3390/ijerph182111507

**Published:** 2021-11-01

**Authors:** Rute Bastardo, Ana Isabel Martins, João Pavão, Anabela Gonçalves Silva, Nelson Pacheco Rocha

**Affiliations:** 1 UNIDCOM, Science and Technology School, University of Trás-os-Montes and Alto Douro, Quinta de Prado, 5001-801 Vila Real, Portugal; rutebastardo@gmail.com; 2 Center for Health Technology and Services Research, Health Sciences School, University of Aveiro, 3810-193 Aveiro, Portugal; anaisabelmartins@ua.pt (A.I.M.); asilva@ua.pt (A.G.S.); 3 INESC-TEC, Science and Technology School, University of Trás-os-Montes and Alto Douro, Quinta de Prado, 5001-801 Vila Real, Portugal; jpavao@utad.pt; 4 Department of Medical Sciences, IEETA-Institute of Electronics and Informatics Engineering of Aveiro, University of Aveiro, 3810-193 Aveiro, Portugal

**Keywords:** older adults, Ambient Assisted Living, usability, usability evaluation, systematic review

## Abstract

This study aimed to determine the methodological quality of user-centered usability evaluation of Ambient Assisted Living (AAL) solutions by (i) identifying the characteristics of the AAL studies reporting on user-centered usability evaluation, (ii) systematizing the methods, procedures and instruments being used, and (iii) verifying if there is evidence of a common understanding on methods, procedures, and instruments for user-centered usability evaluation. An electronic search was conducted on Web of Science, Scopus, and IEEE Xplore databases, combining relevant keywords. Then, titles and abstracts were screened against inclusion and exclusion criteria, and the full texts of the eligible studies were retrieved and screened for inclusion. A total of 44 studies were included. The results show a great heterogeneity of methods, procedures, and instruments to evaluate the usability of AAL solutions and, in general, the researchers fail to consider and report relevant methodological aspects. Guidelines and instruments to assess the quality of the studies might help improving the experimental design and reporting of studies on user-centered usability evaluation of AAL solutions.

## 1. Introduction

The worldwide population is ageing and the related longer life-expectancy represents an extraordinary challenge in terms of public healthcare policies, due to the changing patterns of disease, the demanding expectations of patients, and the financial restrictions. The current ideal political paradigm, supported in concepts such as ageing in place [1] or active ageing [2,3], consider that older adults should continue living in the community rather than being forced to move to residential care units because of their cognitive and physical limitations.

The success of this approach depends not only on the characteristics of the individuals and their health conditions, influenced by different factors that interact with each other continuously and in subtle ways [4], namely physical, mental, and behavioral factors, but also environmental factors (e.g., the living environment, the support of relatives, the availability and accessibility and of health care, social services, or community support [5]). Therefore, innovative solutions are required to guarantee the autonomy and independence of the increasing number of older adults within friendly environments.

Ambient Assisted Living (AAL) is one of the resources available to promote age-friendly environments to facilitate the maintenance of typical activities and values of middle age. The AAL paradigm refers to intelligent technologies, products and services embedded in the physical environment and aims to maintain the independence and general quality of life of the individuals as they age, by providing secure and supportive environments, optimizing healthcare provision, namely when in presence of chronic diseases, promoting healthy lifestyles which positively impact physical and cognitive functioning, and facilitating social involvement and active participation in the society [6].

AAL is supported in the technological developments of the last decades that, among other possibilities, increased the capacity to develop and manufacture systems that employ smart components highly integrated and miniaturized [7]. This remarkable development makes possible the vision of Weiser [8] regarding ubiquitous computing by bringing computing devices into everyday life (e.g., integration of computing power and sensing features into anything, including everyday objects like white goods, toys, or furniture), in such a way that the users would not notice their presence. On the other hand, the AAL shares with ubiquitous computing the provision of effortless interaction, being context awareness [9] an important feature to allow the adaptation of the environment states to the human being preferences.

AAL must have the capacity to properly distinguish the human beings present in the environment, to recognize the individual roles, needs, preferences and limitations, to evaluate situational context, to allow different answers according to personal requirements and situational contexts and to anticipate desires and needs without conscious mediation. Therefore, the aggregation and processing of sensory data from different devices [7], to automatic change the environment are crucial issues of the AAL paradigm [10,11]. 

Moreover, a distinctive characteristic of ubiquitous computing and consequently of AAL is the interaction with all kinds of elements through different types of interfaces. In addition to the well-known graphical user interfaces, other types of interfaces are being proposed combining several input modalities, such as, voice, haptic, gesture or body movement interaction. These interfaces represent an increased diversity in terms of communication channels [12]. Since each independent channel is called a modality, the interaction might be unimodal or multimodal. Multimodal interactions together with context awareness imply high complexity in terms of implementation of user interaction mechanisms, but this high complexity implementation must be translated in simple and usable interfaces. 

The term usability is related to the ability of a product or a service to help the user achieve a specific goal in a given situation while enjoying its use [13,14]. Good usability is usually associated with [15]: lower error rates, more intuitive products and systems, higher acceptance rates and decreased time and effort to attain a specific goal. 

The usability evaluation is an important part of the overall development of user interaction systems, which consists of iterative cycles of design, prototyping and validation. Most development processes focus entirely on adherence to technical specifications. This is one of the main reasons why some products or systems have failed to gain broad acceptance [15]. The introducing of user-centered methods aims to ensure the acceptance of the products and services being developed. 

The literature describes several methods, procedures, and instruments to evaluate the usability of digital solutions [16]. Certain evaluations rely on usability experts (i.e., involving the inspection of the digital solution by experts to evaluate the various aspects of user interaction against an established set of principles of interface design and usability [17,18]), while others rely on end users (i.e., experiments involving end users to determine their perceptions [19]). These perceptions are gathered using different methods (e.g., test and inquiry) and techniques (e.g., interviews, think-aloud, and observation), which are usually combined [20] to perform a comprehensive evaluation. 

Previous reviews aimed to study various aspects of AAL, including technological ecosystems [21], systems architectures [22], human activities’ recognition [23], acceptance in rehabilitation [24], questionnaires for user experience assessment [12], interventions [25,26], and bibliometric analysis [27]. However, the authors of this review were not able to identify studies systematically reviewing and evaluating the evidence on the quality of the user-centered usability evaluation of AAL solutions. Therefore, this systematic literature review aims to (i) identify the characteristics of the AAL studies reporting on user-centered usability evaluation, (ii) systematize the methods, procedures and instruments being used, and (iii) verify if there is evidence on a common understanding on methods, procedures, and instruments for user-centered usability evaluation.

The study intends to contribute to the quality of user-centered usability evaluation of AAL solutions by (i) reviewing the main research recently published, (ii) determining and discussing the usability evaluation methods, procedures, and instruments being used, (iii) determining the major methodological drawbacks, (iv) identifying good practices, and (vi) promoting a common understanding of the methodological approaches. 

## 2. Materials and Methods

This systematic review followed the guidelines of the Preferred Reporting Items for Systematic Reviews and Meta-Analyses (PRISMA) [28]. To perform the systematic literature review, the authors defined a review protocol with explicit descriptions of the methods to be used and the steps to be taken [29]: (i) the research questions; (ii) the search strategies; (iii) the inclusion and exclusion criteria; (iv) the screening procedures; (v) data extraction; (vi) methodological quality assessment; and (vii) synthesis and reporting.

### 2.1. Research Questions

Based on the analysis of the literature in the field of usability evaluation of digital solutions and previous work of the research team, a lack of consensus in the academic literature regarding the methods, procedures, and instruments being used for evaluating usability of AAL solutions was identified. To have a more in-depth knowledge of the practices on user-centered usability evaluation of AAL solutions, the following research question was formulated:

- RQ1: What is the methodological quality of user-centered usability evaluation of AAL solutions?

This broad question was subdivided into three additional secondary research questions:

- RQ2: What are the characteristics of the AAL studies reporting on user-centered usability evaluation in terms of study demographics, publication date, country of publication, purpose of the AAL reported solution and interaction modalities?
- RQ3: What are the methods (e.g., test methods, inquiry methods or both), procedures (e.g., environment where the usability evaluation is conducted), and instruments being used (e.g., validated instruments or purposively developed instruments)?
- RQ4: Do existing studies on user-centered usability evaluation of AAL solutions follow quality recommendations when assessed against the Critical Assessment of Usability Studies Scale (CAUSS) [30]?

### 2.2. Search Strategies

The resources chosen for the review were three electronic databases (i.e., Scopus, Web of Science, and IEEE Xplorer). Boolean queries were prepared to include all the articles that have their titles, abstract or keywords conformed to the conjunction (i.e., AND Boolean operator) of the following expressions:

- “AAL”, “ambient assisted living”, “ambient assisted technology”, “ambient assistive technology” or “ambient intelligence”;
- “UX”, “user experience”, or “usability”;
- “Evaluation” or “assessment”.

The expressivity of the search procedure depends on the database. As an example, the query expression to retrieve articles from the Scopus database was de following: TITLE-ABS-KEY ((AAL or “ambient assisted living” or “ambient assisted technology” or “ambient assistive technology” or “ambient intelligence”) and (UX or “user experience” or usability) and (evaluation or assessment)).

The electronic literature search was performed in January 2021 and included all the references published before 31 December 2020.

### 2.3. Inclusion and Exclusion Criteria

References were included if they reported on user-centered usability evaluation of AAL solutions that might be used to support older adults by promoting secure and supportive environments, optimizing healthcare provision, promoting healthy lifestyles, and facilitating social involvement and active participation in the society [6].

References were excluded if they (i) did not have abstracts, (ii) were not written in English, (iii) reported on reviews, surveys, or market studies, (iv) were books, reported on workshops, or special issues announcements, (v) reported on studies whose primary objectives were not usability assessment, or (vi) reported on studies that were not relevant for the objective of this systematic review.

### 2.4. Screening Procedures

The analysis and selection of the studies were performed in three steps:

- First step—the authors removed the duplicates, the articles without abstract and not written in English;
- Second step—the authors assessed all titles and abstracts for relevance and those clearly not meeting the inclusion and exclusion criteria were removed;
- Third step—the authors assessed the full text of the remaining articles against the outlined inclusion and exclusion criteria and the final list of the studies to be considered for the review was created.

Throughout this entire process, all articles were analyzed by three authors and any disagreement between the authors was discussed and resolved by consensus.

### 2.5. Data Extraction

Concerning data extraction, the following information was registered in a data sheet prepared by the authors for each of the studies included in the review: (i) the demographics of the study (i.e., authors and respective affiliations, year and source of publication); (ii) the scope of the study; (iii) the purpose of the AAL solution being reported; (iv) details of the interaction technologies being used; (v) the methods, techniques, instruments and procedures applied to evaluate usability; (vi) the characteristics of the participants involved in the usability evaluation; and (vi) the outcomes being reported. 

### 2.6. Methodological Quality Assessment

Three authors independently assessed the methodological quality of included studies using a scale developed to assess the methodological quality of studies evaluating usability of electronic health products and services, the Critical Assessment of Usability Studies Scale (CAUSS) [30]. The CAUSS has 15 items that can be scored “yes” or “no”. This scale is both valid and reliable (Intraclass Correlation Coefficient—ICC = 0.81) [30]. Each study was assessed by at least two authors. This quality assessment was undertaken in two steps: first three manuscripts were assessed by all the three authors involved in this step of the review to foster a common understanding of the scale items. Then, all the remaining manuscripts were independently assessed by two of the three authors. During both steps, disagreements were resolved by discussion and a final decision achieved by consensus. Percentage of agreement between the assessors was calculated for each one of the 15 items of the scale.

### 2.7. Synthesis and Reporting

Based on the demographic data of the included studies, a synthesis of studies’ characteristics was prepared, which included: (i) the number of studies published in conference proceedings and in scientific journals; (ii) the distribution of the studies by year and the publication rate, which was calculated using RMS Least Square Fit; and (iii) the distribution of the studies by country. Since some studies involved multidisciplinary teams, it was considered the institutional affiliation of the first author of each study to determine the number of studies per nation.

The different AAL solutions described by the included primary studies were coded in terms of AAL domains and interaction modalities. In what concerns the AAL domains, a tabular presentation was prepared, which considered four domains [6]: (i) secure and supportive environment; (ii) healthcare provision; (iii) healthy lifestyles; (iv) social involvement and active participation. These domains were further divided into various purposes [6]: (i) daily living activities and falls prevention for secure and supportive environment; (ii) home monitoring, remote care, telerehabilitation, and medication management for healthcare provision; (iii) physical activity, cognitive activity, physical and cognitive activity for healthy lifestyles; and (iv) social inclusion and participation in leisure activities for social involvement and active participation. In turn, concerning the interaction modalities, the classification considered both the traditional unimodal graphic user interface approach (i.e., visual interaction) and multimodal approaches (i.e., visual interaction together with voice, auditory, gesture or other interaction modalities, such as immersive virtual reality or robots) [12]. 

In terms of usability evaluation, the number and mean age of the participants, as well as the testing environment were identified, and the procedures used in each study were classified into test and inquiry methods and respective techniques: the method of test includes techniques such as observation, performance or think aloud and the method of inquiry includes techniques such as interviews, scales, or questionnaires. 

Finally, based on the results of the application of CAUSS, the authors performed an analysis of the usability evaluation methods, procedures, and instruments of the included studies and a tabular and narrative synthesis was prepared.

## 3. Results

### 3.1. Study Selection

**Figure 1:**
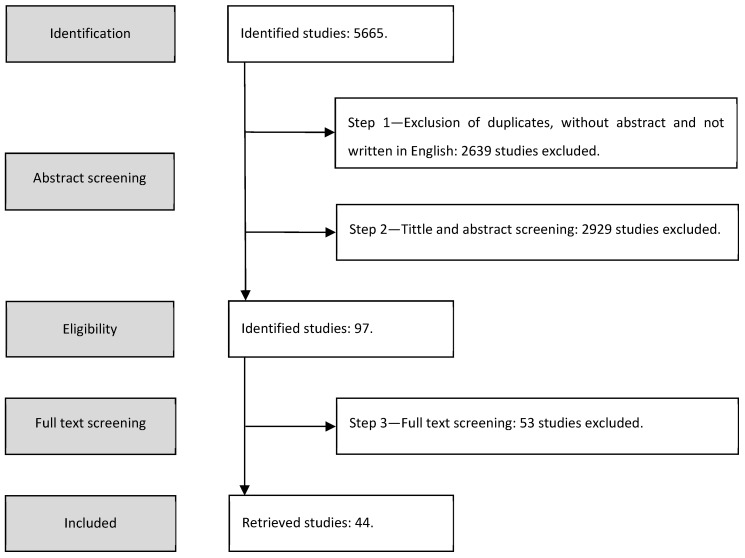
Systematic Reviews Flowchart.

Figure 1 presents the flowchart of the systematic review. A total of 5635 studies were retrieved from the initial search of the selected databases. 

The first step yielded 3026 studies since 2639 studies were removed because they (i) were duplicated (i.e., 874 studies), (ii) did not have abstracts (i.e., 1734 studies), or (iii) were not written in English (i.e., 31 studies).

During the second step, one study was excluded because it was retracted and 2928 studies were removed, because they (i) reported on reviews, surveys, or market studies (i.e., 234 studies), (ii) were books, reported workshops, or were special issues announcements (i.e., 98 studies), or (iii) were not relevant for the objective of this systematic review, since they did not report user-centered usability evaluation of AAL solutions that might be used to support older adults (i.e., 2596 studies).

Finally, after the full text analysis (i.e., the third step), 53 studies were removed since they did not meet the inclusion and exclusion criteria. 

Therefore, 44 studies [31,32,33,34,35,36,37,38,39,40,41,42,43,44,45,46,47,48,49,50,51,52,53,54,55,56,57,58,59,60,61,62,63,64,65,66,67,68,69,70,71,72,73,74] were included in this systematic review.

### 3.2. Demographics of the Included Studies

Of the included 44 studies, some reported on the same research projects: studies [32,33], studies [45,46,47] and studies [36,52,58] were respectively related to the European funded projects ALADIN, iStoppFalls and Robot-ERA, while [68,74] were related to a project funded by the European Commission and co-funded by the Swiss Confederation.

In terms of publication types, ten studies were published in conference proceedings [31,32,34,35,38,40,42,61,62,72] and 34 studies were published in scientific journals [33,36,37,39,41,43,44,45,46,47,48,49,50,51,52,53,54,55,56,57,58,59,60,63,64,65,66,67,68,69,70,71,73,74].

**Figure 2:**
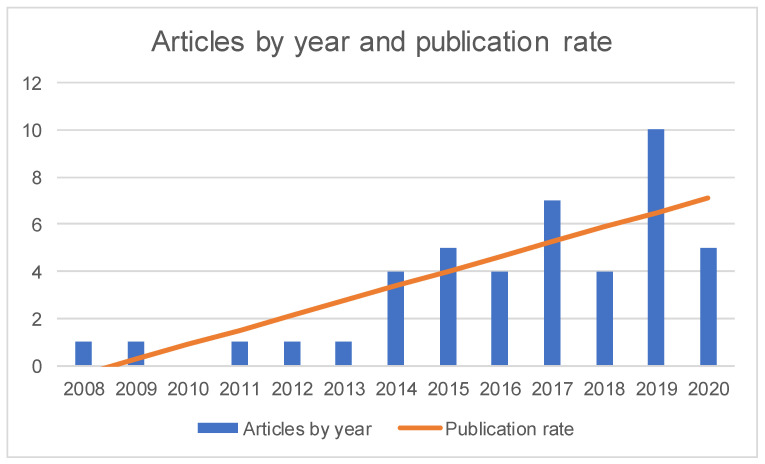
Studies by year and publication rate (calculated using RMS Least Square Fit).

Concerning the publication years, the included studies were published between 2008 (i.e., one study [31]) and 2020 (i.e., five studies [70,71,72,73,74]). The diagram in Figure 2 demonstrates a trend towards an increasing number of publications, and more than two-thirds of the studies (i.e., 30 studies [45,46,47,48,49,50,51,52,53,54,55,56,57,58,59,60,61,62,63,64,65,66,67,68,69,70,71,72,73,74]) were published in the last five years and more than one-third of the studies (i.e., 15 studies [60,61,62,63,64,65,66,67,68,69,70,71,72,73,74]) were published in the last two years.

**Figure 3:**
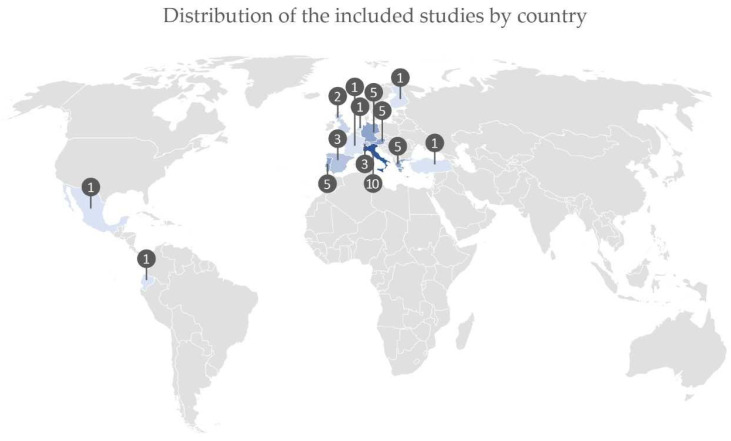
Distribution of the selected studies by country.

The Figure 3 represents the distribution of the included studies by country. Europe has the highest contribution (i.e., 43 studies). Comparatively, the remaining regions of the world have relatively residual contributions: together, North America, South America and Asia contributed with three studies.

As can be seen in Table 1, 16 studies (i.e., 36% of the included studies) reported on the involvement of multinational research teams.

### 3.3. Purpose of the Reported AAL Solutions

Table 2 presents the AAL domains and purposes of the included studies. The promotion of secure and supportive environments and the optimization of healthcare provision were the AAL domains with the highest number of studies, respectively 20 and 14 studies.

From the 20 studies related to secure and supportive environments, 15 were focused on AAL solutions to support daily living activities and, consequently, to increase the independence of older adults, while five studies were focused on AAL solutions to prevent falls. 

In turn, the 14 studies related to healthcare provision were focused on home monitoring (five studies), telerehabilitation (four studies), remote care (three studies) and medication management (two studies).

Moreover, eight studies reported on usability evaluation of AAL solutions to promote healthy lifestyles (i.e., physical and cognitive activities). Finally, two studies reported on the usability evaluation of AAL solutions to promote social involvement and active participation. One of the studies was focused on social inclusion, while the other reported the use of AAL solutions to promote the participation of older adults in leisure activities. 

### 3.4. Interaction Modalities

Concerning the interaction modalities (Table 3), 11 studies reported on unimodal approaches based on visual interaction. In turn, the remainder studies reported on multimodal approaches based on different interaction technologies, namely visual interaction together with voice, auditory, gesture, or other interaction modalities, such as immersive virtual reality or robots.

### 3.5. Methodological Quality Assessment

**Figure 4:**
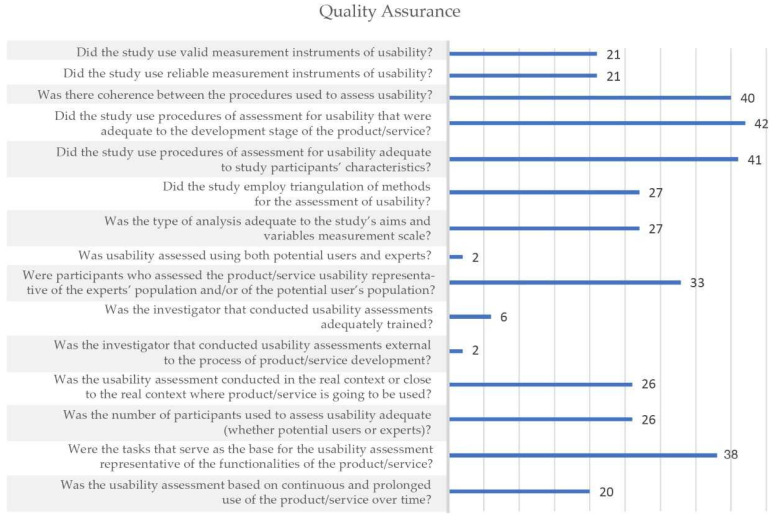
Number of studies that met each item, after consensus was reached between reviewers.

According to Figure 4, only four items of the methodological quality scale were scored positively for more than 80% of included studies (i.e., the items 3, 4, 5, and 14). In contrast, there are three items that were scored positively for only 6 or less (≤13%) of the included studies (i.e., the items 8, 10, and 11). All the remaining items (i.e., eight items) were scored positively for 20 (44%) to 33 (72%) of included studies. Percentage agreement between the two authors who performed the quality ratings of each item varied between 78% and 96% (Table 4). Moreover, the percentage of agreement between each one of three pairs of evaluators considering the different items were similar and varied between 71% and 100%. 

### 3.6. Detailed Analysis of Usability Evaluation Procedures

Details of the experimental studies of the included studies are present in Table 5, namely the solution being evaluated, the usability assessment methods and techniques that were used, number and average age of the participants and the test environments. 

A detailed analysis of usability evaluation procedures (Table 6) revealed that test methods were used in 25 studies and inquiry methods were used in 39 studies. These add up to more than the number of included studies (i.e., 44), as 20 (45%) studies were based on a multimethod approach (i.e., combined both test and inquiry methods). Among the different techniques of the test method, the most reported were observation (*n* = 13; 29%) and performance evaluation (*n* = 12; 27%). Regarding inquiry, the most reported techniques were questionnaires/scales (*n* = 35; 80%) and interviews (*n* = 13; 29%). Several studies combined two or more techniques of the same method. Of the 35 studies that used scales/questionnaires, 19 (54%) studies used at least one valid and reliable usability evaluation instrument, 13 (37%) used questionnaires developed by the authors of the included studies without any reference to their psychometric characteristics (e.g., validity and reliability) and three (9%) used instruments based on technology acceptance models (Table 7). 

In terms of valid and reliable instruments (Table 7), the System Usability Scale (SUS) emerged as the most used instrument, being used in 16 studies (i.e., [43,45,54,56,58,59,61,64,65,66,67,68,69,70,71,74]). Other instruments being used, both alone or in combination with SUS, were the IBM Computer Usability Satisfaction Questionnaires (IBM-CUSQ) (*n* = 2), the ICF-Based Usability Scale (ICF-US) (*n* = 2), the Post-Study System Usability Questionnaire (PSSUQ) (*n* = 1), the Usefulness, Satisfaction and Ease of Use Questionnaire (USE) (*n* = 1), and the Human Robot Interaction Scale (HRI) (*n* = 1).

With regards to model-based instruments, the models used were the Technology Acceptance Model (TAM) (*n* = 2) and the Unified Theory of Acceptance and Use of Technology (UTAUT) (*n* = 1).

Each study number of participants varied from four (i.e., studies [37,42]) to 153 (i.e., study [45]). Two studies did not include any information related to the age of the participants [60,72]. In turn, six studies did not report the mean age of the participants, although in five of them participants were at least 65 years old [32,39,48,55,67], while in another study, participants were at least 50 years old [66]. Considering the studies that reported the mean age of participants, in three of them [40,59,73] the mean age was less than 60 years of age, while in the remainder of the studies, the mean age was higher than 60 years old.

The environment where usability evaluation was conducted is diverse (Table 8). For 13 out of the 44 included studies (30%), usability evaluation was conducted at the participant’s homes, in 10 (23%) studies it was conducted at institutional sites as day care or nursing homes, in 12 (27%) studies it was performed in a living lab, and in nine studies (20%) it was conducted at research labs.

## 4. Discussion

This systematic review aimed to synthetize existing evidence on user-centered usability evaluation of AAL solutions. 

Concerning the characteristics of the AAL studies reporting on user-centered usability evaluation (i.e., the second research question), the domains and purposes of the AAL solutions described in the included publications are in line with the AAL program objectives and include the promotion of secure and supportive environments and the optimization of healthcare provision, promotion of healthy lifestyles and promotion of social involvement and active participation [75]. Concerning the interaction modalities, most publications report on solutions with multimodal approaches based on different interaction technologies, namely visual, voice, auditory and gesture interaction. Multimodality is a critical factor in the successful deployment of AAL solutions [76] enabling individuals with different needs or the same individual in different contexts to select specific interaction modes.

Results also show that there is a growing trend of interest in the usability evaluation of AAL solutions, which is reflected in the increasing number of publications over the last years. Most authors are affiliated at Institutions based in Europe, which is a predictable result, as the AAL emerged as an initiative of the European Union that aimed to respond to the needs of the elderly population in Europe [75,77]. However, although the AAL program aims to create synergies between researchers based at different European countries, the research teams of most publications are affiliated to institutions from the same country, suggesting that this aim of the AAL program was not fully accomplished. Moreover, despite the strong investment of European Commission and Member States, it seems that the AAL concept has a poor expressiveness outside Europe.

Considering the methods, procedures, and instruments being reported (i.e., the third research question) more than half of the studies of this review used only one usability evaluation method, being the inquiry method the most used. An important aspect in usability evaluation is to use valid and reliable evaluation instruments (i.e., scales and questionnaires). However, in this review, many studies reported on the utilization of ad-hoc instruments. Examples of poor practices from a methodological quality point of view are the use of questionnaires developed purposively for a study without any attempt to assess its validity by an expert panel or against a gold standard and without specifying the questions and the process of development (e.g., [35,36,38,39,41,49,55,63,73]), extracting some questions from previously validated instruments compromising their validity and reliability (e.g., [53]), or assessing reliability but not validity (e.g., [52,60]). Ensuring validity is ensuring that an instrument is assessing what is supposed to be assessed and ensuring reliability is ensuring that the instruments give consistent results across repeated assessments. Although there might be reasons to develop or adapt a scale/questionnaire, its validity and reliability must be evidenced [77], which was not the case of the questionnaires used in 37% of the studies included in this review (i.e., [35,36,38,39,41,49,52,53,55,60,63,73]). The finding that SUS was the most commonly usability scale reported in the included studies (i.e., [43,45,54,56,58,59,61,64,65,66,67,68,69,70,71,74]) is in line with a previous review on user-centered usability evaluation [78] and it suggests that this is a widely accepted instrument, usually regarded as a golden standard in terms of usability evaluation. 

In this review, most studies were conducted either in real environments (e.g., participant’s homes or institutional sites as day care or nursing homes) or in conditions that simulate the home environment (i.e., living labs). Only 12 studies were carried out in the laboratory context, but it is not possible to establish an association between the testing environment and the maturity level of the applications (e.g., the solution reported by [32] was in an early development stage and the usability evaluation was carried out in an institutional context) nor between the testing environment and the purposes of the applications being developed. For instance, the seven studies evaluating the usability of social robots were conducted in living labs [34,36,38,57,58], institutional site [71] and participant’s home [49], while an application to support daily tasks [69] was evaluated in a laboratory context. Moreover, among the five applications aiming to prevent falls [45,46,47,48,66], only one was evaluated in a laboratory context [66], although all these applications had equivalent maturity levels. Similarly, among the four included telerehabilitation applications [44,56,62,63], one application [63] was evaluated in an institutional site, although its maturity level was identical to the maturity level of applications with identical purposes that were evaluated in a laboratory context (i.e., [44,56]). In turn, among the four applications proposing virtual physical training solutions [35,54,61,64], the one that was evaluated in a research laboratory (i.e., [61]) was in an early development stage when compared with the other three applications. 

Thirty-two studies (73% of the included studies) were carried out in the participants’ home, institutional sites and living labs. This suggests a concern of studies’ authors for taking the evaluation closer to real conditions, even for applications in an early development stage (e.g., [32]).

A limited number of studies present both usability evaluation by users and by experts. Combining these two types of evaluation is recommended as a good practice to have a comprehensive and complementary view of potential usability problems [79]. However, this result might be biased due to the focus of the present review on user-centred usability evaluation.

Whether the researcher conducting the usability evaluation received adequate training or is external to the team of researchers who developed the AAL solution is seldom reported by the studies included in this review. However, this information is of great relevance as both the inexperience of the researcher and a potential conflict of interest might impact the results of the usability evaluation [80]. Usability evaluation involves close interaction between the researcher and the participants, methods and procedures are complex and depend on this interaction and, therefore, require experience and knowledge to be assessed effectively as well as independence to minimize the potential for unwantedly influencing participants [80].

Considering the first and primary research question (i.e., what is the methodological quality of user-centered usability evaluation of AAL solutions?), the results of this review suggest that there is the need to pay careful attention to quality as a considerable number of studies fail to report on pre-identified quality criteria. Nevertheless, these findings are aligned with the findings of previous reviews using the same quality scale, the CAUSS [30,78]. 

The development of scientific knowledge is built on existing knowledge and new research to achieve a deeper understanding of a particular topic. As in any another scientific topic, the generation of comprehensive knowledge related to the usability of AAL solutions depends on the methodological quality of the respective research studies. The lack of robust methodological approaches prevents the generalization of research results and the consolidation of the area.

Specifically, the results of this review demonstrate that the methodological quality of user-centred usability evaluation of AAL solutions prevents not only the generalization of results to be used and deepened in further studies, but also the translation of the developed solutions. In this respect, it should be pointed that some AAL solutions might be considered as medical devices, which means that their translation to daily use solutions requires a certification according to very strict regulatory frameworks that consider as mandatory requirement the high methodological quality of all assessment procedures [81]. Moreover, usability evaluation is only one step of all the assessment steps that must be performed. For instance, once it has been demonstrated that an AAL product or service is usable, there is the need of experimental studies involving end users to assess the efficacy and efficiency of the proposed solutions. This means that unreliable results in terms of usability evaluation might have consequences for subsequent assessments.

Looking for the results of the AAL European strategy, although a considerable investment was made for creating market-ready products and services for older people, and the significant number of projects, only a residual number of solutions reached the market [27,82]. This is a consequence of multiple causes, including the difficulty in tailoring AAL solutions to individual end-users’ needs [82], which demands not only deep knowledge and comprehensive definitions of user requirements, but also robust methodologies to assess the feedback of the end-users. Furthermore, inefficiencies of the research process that facilitates the propagation of mistakes and promotes useless and costly repetition represent huge barriers for the translation of results into innovation [83].

Based on the analysis of the included studies, it is possible to conclude that there is heterogeneity on the methods, procedures, and instruments for the user-centered usability evaluation. The validity and reliability of scientific results are essential for their reproducibility and reusability, namely for creating market-ready solutions. Therefore, to facilitate the reproducibility and reusability of the research results related to AAL solutions the openness and the transparency of all steps of the research process should be increased. Among other requirements, there is a need to comply with established methodological guidelines, standardized study designs, and use of reporting checklists to ensure a detailed description of study methods and resulted data [83]. An example of guidelines that can be used to inform both study design and study reporting, in addition to the CAUSS which was used in the present study as a guide for methodological quality assessment, is suggested by our team in a previous publication [80]. This proposed a guide to consider when designing and reporting a user-centered usability evaluation study and includes aspects such as the characteristics of the person conducting the usability evaluation that should be reported, characteristics of the participants assessing the digital solution that should be reported, aspects of methods and techniques used and environment where the usability evaluation is taking place. By using these guidelines at the designing stage authors guarantee that methodological options are carefully considered in advance and by using them at the reporting stage authors improve reproducibility and comparability of results across studies. Improving the reporting of usability evaluation studies will also facilitate, in the long term, the comparison of the ability of different usability procedures in detecting usability problems.

The results of this review should be viewed against some limitations, mainly related to the search strategy. Defining the search keywords was problematic due to many terms employed by researchers. Moreover, although the databases that were considered (i.e., Scopus, Web of Science, and IEEE Xplorer) are representative of the scientific literature, probably there are similar studies that were not indexed by these databases. Furthermore, the reliance solely on the English language may reduce the number of studies considered in the analysis. Finally, grey literature was not considered.

Despite these limitations, in terms of research methods, the authors tried to follow rigorous procedures for the studies’ selection and data extraction, so that the results of the evaluation of methodological quality of user-centered usability evaluation of AAL solutions are relevant and might contribute to the quality of future studies.

## 5. Conclusions

An overall analysis of the results suggests that there is high heterogeneity among user-centered usability evaluation studies in terms of methods, procedures, and instruments. Furthermore, studies are of low methodological quality as they fail to consider and report relevant methodological aspects. Therefore, as a main conclusion of this study, future AAL research must pay special attention to the design and reporting of the studies on user-centered usability evaluation.

In this respect, guidelines and instruments to assess the quality of the studies, such as CAUSS [30], which has been used for the methodological quality assessment of the included studies, should be considered when designing and reporting user-centered usability evaluations of AAL solutions. 

## Figures and Tables

**Table 1:** Multinational teams.

References	Multinational Teams
[33]	Germany, United States of America, Italy, Austria, and Romain
[35]	Germany, Sweden, and France
[36]	Italy and Sweden
[37]	Finland and Spain
[39]	Spain and United Kingdom
[41]	Portugal and Spain
[47]	Austria, Australia, and Chile
[49]	Greece, Italy, and United Kingdom
[53,56]	Italy and Spain
[58]	United Kingdom, Italy, and Belgium
[63]	France and Greece
[64,68]	Switzerland and Sweden
[67]	Netherlands and Spain
[71]	Greece, Germany, Italy, and United Kingdom

**Table 2:** Domains and purposes of the AAL solutions reported by the included studies.

Domains	Purposes	References
Secure and supportive environment	Daily living activities	[31,32,33,34,36,38,39,42,49,57,58,59,60,69,71]
	Falls prevention	[45,46,47,48,66]
Healthcare provision	Home monitoring	[41,43,65,67,70]
	Telerehabilitation	[44,56,62,63]
	Remote care	[40,52,55]
	Medication management	[37,50]
Healthy lifestypes	Physical activity	[35,54,61,64,73]
	Cognitive activity	[72]
	Physical and cognitive activity	[68,74]
Social involvement and active participation	Social inclusion	[51]
	Participation in leisure activities	[53]

**Table 3:** Interaction modalities and respective terminal equipment.

Interaction	Terminal Equipment	References
Visual Interaction	Personal computer	[66]
	Mobile (i.e., tablet or smartphone)	[60,62,65,72]
	Mobile and personal computer	[41]
	Mobile and interactive TV	[54]
	Interactive TV	[32,33,40,49]
Visual and auditory interaction	Mobile	[37,51]
	Interactive TV	[53,55,70]
Visual and voice interaction	Mobile	[43]
Visual, voice and auditory interaction	Mobile	[42,50,67]
	Mobile and interactive TV	[39]
	Enhanced communication agents	[31,35]
Visual and gesture interaction	Personal computer and WiiMote	[44]
	Personal computer and Kinect	[61]
	Interactive TV and wearable inertial sensors	[68,74]
	Interactive TV, wearable inertial sensors and Kinect	[45,46,47]
	Interactive TV and Kinect	[73]
	Interactive TV and position sensors	[64]
	Personal computer, RGB cameras and depth sensors	[63]
Other interaction modalities	Immersive virtual reality	[56,69]
	Robots	[34,36,38,48,52,57,58,59,71]

**Table 4:** Level of agreement between the reviewers for each item of CAUSS.

Item	1	2	3	4	5	6	7	8	9	10	11	12	13	14	15
Agreement	78%	78%	98%	93%	87%	80%	78%	83%	89%	85%	91%	87%	85%	96%	78%

**Table 5:** Usability assessment design.

#	Solution Being Evaluated	Test				Inquiry				Participants		Test Environment			
		P ^1^	O ^2^	T ^3^	W ^4^	I ^5^	S ^6^	Q ^7^	C ^8^	Number	Mean Age (Years)	I ^9^	L ^10^	P ^11^	R ^12^
[31]	Smart companion	-	-	-	x	-	-	-	-	10	82	x	-	-	-
[32]	Adaptive lighting application	-	x	-	-	-	-	-	-	12	- ^13^	x	-	-	-
[33]	Adaptive lighting application	-	x	-	-	-	-	-	-	12	71	x	-	-	-
[34]	Social robot	-	-	x	-	-	-	x	-	16	77	-	x	-	-
[35]	Virtual physical training	-	-	-	-	x	-	x	-	30	69	x	-	-	-
[36]	Social robot	-	x	-	-	-	-	x	-	25	74	-	x	-	-
[37]	Medication management	-	x	-	-	x	-	-	-	4	80	x	-	-	-
[38]	Social robot	-	-	-	-	-	-	x	-	15	74	-	x	-	-
[39]	Smart Kitchen	-	x	-	-	-	-	x	-	63	- ^13^	-	x	-	-
[40]	Remote care	-	x	-	-	-	-	-	-	30	58	x	-	-	-
[41]	Home monitoring	-	-	-	-	-	-	x	-	11	70	-	-	x	-
[42]	Smart companion	x	-	-	-	-	x	x	-	4	80	-	x	-	-
[43]	Home monitoring	x	x	x	-	-	x	x	-	25	76	-	-	x	-
[44]	Telerehabilitation	-	x	-	-	-	-	x	-	32	65	-	-	-	x
[45]	Falls prevention	-	x	-	-	x	x	-	-	153	73	-	x	-	-
[46]	Falls prevention	-	-	-	-	x	-	-	-	12	73	-	-	x	-
[47]	Falls prevention	-	-	-	-	x	-	-	-	62	74	-	-	x	-
[48]	Falls prevention	-	-	-	-	x	-	-	-	14	- ^13^	-	-	x	-
[49]	Social robot	-	-	-	-	x	x	x	-	7	79	-	-	x	-
[50]	Medication management	-	x	x	-	-	x	x	-	10	70	-	-	-	x
[51]	Application to promote social inclusion	x	-	-	-	x	-	x	-	22	66	-	-	x	-
[52]	Remote care	-	-	-	-	-	-	x	-	23	73	-	x	-	-
[53]	Leisure activities	x	-	-	-	-	-	x	-	20	70	-	-	x	-
[54]	Virtual physical training	-	-	-	-	-	x	-	x	14	73	-	x	-	-
[55]	Remote care	-	-	-	-	-	-	x	-	62	- ^13^	-	-	x	-
[56]	Telerehabilitation	-	-	-	-	x	x	-	-	5	70	-	-	-	x
[57]	Social robot	-	-	-	-	-	-	x	-	17	75	-	x	-	-
[58]	Social robot	-	-	-	-	-	x	x	-	82	78	-	x	-	-
[59]	Social robot	-	-	-	-	-	x	-	-	25	37	-	-	-	x
[60]	Safety application	-	-	-	-	-	-	x	-	44	- ^13^	-	x	-	-
[61]	Virtual Physical training	x	-	-	-	x	x	-	-	12	73	-	-	-	x
[62]	Telerehabilitation	x	x	-	--	-	-	-	-	4	72	-	-	-	x
[63]	Telerehabilitation	-	-	-	-	x	-	x	-	6	80	x	-	-	-
[64]	Virtual physical training	x	-	x	-	-	x	-	-	12	72	x	-	-	-
[65]	Home monitoring	x	-	-	-	-	x	x		10	80	x	-	-	-
[66]	Falls prevention application	x	-	x	-	x	x	-	-	15	- ^13^	-	-	-	x
[67]	Home monitoring	-	-	-	-	-	x	x	-	26	- ^13^	-	-	x	-
[68]	Virtual physical and cognitive training	x	x	x	-	-	x	x	-	21	74	-	-	x	-
[69]	Support to daily tasks	-	-	-	-	x	x	-	-	6	60	-	-	-	x
[70]	Home monitoring	-	-	-	-	-	x	-	-	19	73	-	-	x	-
[71]	Robot	x	-	-	-	-	x	-	-	104	74	x	-	-	-
[72]	Cognitive game	-	-	-	-	-	-	x	-	63	- ^13^	x	-	-	-
[73]	Virtual physical training	x	-	-	-	-	-	x	-	135	46		x	-	-
[74]	Virtual physical and cognitive training	-	x	x	-	-	x	x	-	21	71	-	-	-	x

Notes: x reported; - not reported; Test method (^1^ Performance; ^2^ Observation; ^3^ Think aloud; ^4^ Wizard of Oz); Inquiry method (^5^ Interviews; ^6^ Scales; ^7^ Questionnaires; ^8^ Card sorting); Test environment (^9^ Institutional site; ^10^ Living lab; ^11^ Participant home; ^12^ Research lab); Participants (^13^ Mean age of the participants not reported).

**Table 6:** Usability evaluation methods.

Methods	Studies
Exclusively test methods	[31,32,33,40,62]
Exclusively inquiry methods	[35,38,41,47,48,49,52,54,55,56,57,58,59,60,63,67,69,70,72]
Multimethod (test and inquiry methods)	[34,36,37,39,42,43,44,45,46,50,51,53,61,64,65,66,68,71,73,74]

**Table 7:** Usability evaluation instruments.

Instruments Nature	Study
Validated scales and questionnaires	[34,42,43,45,50,54,56,58,59,61,64,65,66,67,68,69,70,71,74]
Ad-hoc scales and questionnaires	[35,36,38,39,41,49,52,53,55,60,63,73]
Scales and questionnaires based on technology acceptance models	[44,51,57,72]

**Table 8:** Environments where the usability evaluations were conducted.

Test Environment	Studies
Participant’s home	[41,43,46,47,48,49,51,53,55,64,67,68,70]
Institutional site as day care or nursing home	[31,32,33,35,37,40,63,65,71,72]
Living lab	[34,36,38,39,42,45,52,54,57,58,60,73]
Research lab	[44,50,56,59,61,62,66,69,74]

## Data Availability

Not applicable.
